# A Novel Multiinstance Learning Approach for Liver Cancer Recognition on Abdominal CT Images Based on CPSO-SVM and IO

**DOI:** 10.1155/2013/434969

**Published:** 2013-12-04

**Authors:** Huiyan Jiang, Ruiping Zheng, Dehui Yi, Di Zhao

**Affiliations:** ^1^Software College, Northeastern University, Shenyang 110819, China; ^2^Key Laboratory of Medical Image Computing of Ministry of Education, Shenyang 110819, China; ^3^Department of Hepatobiliary Surgery, First Affiliated Hospital of China Medical University, Shenyang 110001, China

## Abstract

A novel multi-instance learning (MIL) method is proposed to recognize liver cancer with abdominal CT images based on instance optimization (IO) and support vector machine with parameters optimized by a combination algorithm of particle swarm optimization and local optimization (CPSO-SVM). Introducing MIL into liver cancer recognition can solve the problem of multiple regions of interest classification. The images we use in the experiments are liver CT images extracted from abdominal CT images. The proposed method consists of two main steps: (1) obtaining the key instances through IO by texture features and a classification threshold in classification of instances with CPSO-SVM and (2) predicting unknown samples with the key instances and the classification threshold. By extracting the instances equally based on the entire image, the proposed method can ignore the procedure of tumor region segmentation and lower the demand of segmentation accuracy of liver region. The normal SVM method and two MIL algorithms, Citation-kNN algorithm and WEMISVM algorithm, have been chosen as comparing algorithms. The experimental results show that the proposed method can effectively recognize liver cancer images from two kinds of cancer CT images and greatly improve the recognition accuracy.

## 1. Introduction

With the development of computer technology, computer aided diagnosis (CAD) [1] technology used in quantitative analysis of medical imaging arose at the historic moment and became one of the research hotspots in medical imaging. Imageological diagnosis for liver cancer mainly includes four ways, angiography, ultrasonic scan, computed tomography (CT), and magnetic resonance imaging (MRI). In the early diagnosis of liver cancer, the CT image is generally preferred by the doctor [2] because of its high resolution, low damage to human body, and the ability to reflect the pathological position of liver cancer accurately. In traditional image diagnosis, the diagnosis of a mass of CT images brings a radiologist a huge workload. And an omission of a tiny detail because of the differences of visions or experiences may cause a wrong classification [3]. Moreover, liver cancer has the characteristics of difficult treatment, poor curative effect, and high mortality. So, it urgently needs liver cancer CAD to give advisory opinions to the doctor and help improve the correct diagnostic rate.

Traditional liver cancer recognition methods in CAD can be roughly divided into two categories, learning-based classification and nonparametric classification. The approach of learning-based classification mainly includes Bayesian-based approach [4], SVM-based approach [5, 6], and ensemble learning approaches [7, 8]. In these methods, the classified image is the entire medical image [9], and the input features for classifier are usually from the region of interest (ROI). For example, the ROI of a liver cancer samples is the tumor region. However, the segmentation results of tumor region are always not accurate because the contrast of tumor regions, image artifacts, and other organizations is not obvious. This results in the fact that the tumor features extracted from ROIs are not accurate. Finally, it will have a great influence on classification accuracy.

As for the above-mentioned problems, Hu et al. [10] introduced MIL first to the classification of breast tumors in ultrasound images. Using MIL can more clearly express the image with both tumor region and normal region, so as to solve the problem of multiple ROIs classification. However, Citation-kNN algorithm used in [10] has two problems.

1. Not considering the distribution characteristics of the images, such as relative distance, scattered degree, and sparse degree. It results that the classification accuracy is not high.
2. As a lazy learning algorithm, Citation-kNN needs to save the whole training set and go through the whole sample space when predicting. So it will cost a lot of time when classifying.

MIL was first proposed by Dietterich et al. in the context of drug activity prediction [11]. Since MIL was put forward, a lot of related learning algorithms have been proposed. Maron and Ratan [12] defined diverse density function and proposed Diverse Density (DD) algorithm by seeking optimal point of diverse density function as a concept point in the instances' attribute space. Zhang and Goldman [13] proposed EM-DD algorithm by combining DD algorithm with the Expectation Maximization (EM). Wang and Zucker [14] improved the K-nearest neighbor (kNN) algorithm and proposed two lazy learning algorithms named Bayesian-kNN algorithm and Citation-kNN algorithm. Andrews et al. [15] proposed mi-SVM algorithm and MI-SVM algorithm by introducing the MIL constraints to the objective function of SVM. Gartner et al. put forward MIL kernels, such as set kernels and statistic kernels, which are used to measure the similarity between two bags, and then the MIL problem will be transformed into traditional SVM learning problem. Chen and Wang [16] proposed DD-SVM MIL algorithm through the space conversion method. Zhou and Xu [17] proposed MissSVM algorithm using a special semisupervised SVM for MIL.

Huang [18] studied the combination of SVM and MIL (SVM-MIL) further and proposed an SVM-MIL method named WEMISVM. They converted MIL problem to the traditional single instance learning problem through dissolving of every bag and labeling its instances a consistent value with each bag's label. In the training phase, they regarded the average of the instance possible values calculated by voting method in ensemble learning as the label of the target bag. However, applying WEMISVM method to liver cancer recognition has a big problem. WEMISVM method assumes that the instances in one bag are independent of each other, and each instance has the same influence on its bag's label. While in fact each instance has a different influence on its bag's label, for example, the liver cancer block should have much influence on the label of the liver cancer image than the other blocks. In addition, the classification accuracy of WEMISVM method on 14 data sets is also not very high.

SVM is a supervised classifier which aims at finding hyperplane that separates the dataset with maximum margin [19]. The SVM parameters directly affect the learning ability and generalization ability of the classifier. So the improvement of SVM is usually realized by the optimization of SVM parameters. Recently there are many algorithms for SVM parameters optimization, such as genetic algorithm (GA), ant colony optimization (ACO), and particle swarm optimization (PSO). PSO has a high precision and fast convergence rate, so it is generally used in parameter optimization. However, in this paper, every sample needs to use the parameters optimization once, which means that using this method for parameter optimization will consume a lot of time. local optimization (LO) can reduce the time for optimization when there is a good reference point. So we use a combination algorithm of particle swarm optimization and local optimization (CPSO) to optimize the parameters.

In order to obtain a classifier with high classification accuracy and low time complexity for liver cancer recognition, we use MIL method to solve multiple ROIs classification problem, use the idea of bag dissolution to convert MIL problem into a single instance learning problem, use CPSO-SVM to obtain the label of the target bag, and use ensemble learning method to improve the classification performance, and finally we proposed the SVM-IOMIL algorithm. The advantages of our algorithm are as follows.

1. The instances are extracted equally based on the entire image, so our method can ignore the process of tumor region segmentation and lower the requirements of liver region segmentation accuracy.
2. Through two-time instance optimization to find the key instances and the modified CPSO-SVM classifier to classify, our method greatly improved the recognition accuracy of liver cancer.

The rest of this paper is arranged as follows.  Section 2 gives a simple description of SVM and MIL and a specific description of our proposed algorithm. Results and discussion of our method are presented in Section 3.  Section 4 concludes this paper and expounds our future work.

## 2. Materials and Method

### 2.1. Multiinstance Learning

In the field of machine learning, according to the ambiguity of training data, this field can be roughly divided into three learning frameworks: supervised learning, unsupervised learning, and reinforcement learning. As a new learning framework, MIL [10] is the new weak supervised learning method presented by Dietterich et al. to solve the problem of molecular activity prediction. It can be described as follows.

**Figure 1:**
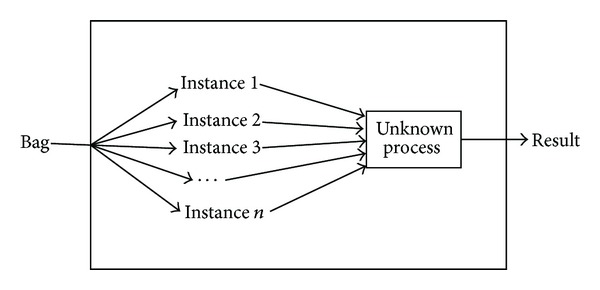
MIL framework diagram.

We suppose that each data in the training data set is a bag, which is a set of instances, and each bag has a training label, while the instances in the bag are not labeled. If a bag is labeled positive, there will be at least one positive instance in the bag. If a bag is labeled negative, all of the instances in it are negative. The goal of MIL algorithm is to train a classifier, which can classify unseen bags correctly by learning the training bags. MIL framework is shown in Figure 1.

### 2.2. Support Vector Machine

In the 1990s, Vapnik [20] proposed SVM theory for solving classification problems. The theory is based on VC dimension theory and structural risk minimization in statistical learning theory (SLT). In order to obtain the best classification performance and promotion capability, the theory uses the information of limited instances to seek the best compromise between the complexities of the model. Since SVM has shown a good learning ability, performance, and the ability of generalization, it causes great attention to the field [21].

SVM is a supervised classifier which aims at finding hyperplane that separates the dataset with maximum margin. Suppose that the training sample set is {(*x*_1_, *y*_1_), (*x*_2_, *y*_2_),…, (*x*_*n*_, *y*_*n*_)}, where *x*_*i*_ ∈ *R* ^*m*^ (*i* = 1,2,…, *n*) stands for the *i*th sample, *n* is the number of training samples, and *y*_*i*_ ∈ {−1,1} is the corresponding category label. Before training, it needs to map the input vector to a high-dimensional feature space *H* using a mapping function. Then, in this high-dimensional space, it needs to construct hyperplane which has the largest classification interval, namely, the optimal hyperplane, to ensure minimum classification error rate.

The classification surface equation is *w* · *z* + *b* = 0, and then we obtain a mapping Φ : *R*_*m*_ → *H*. The objective function is shown as

(1) $$\begin{array}{c} \min L\left(w\right)=\frac{1}{2}{||w||}^{2}+C\overset{N}{\underset{i=1}{\sum }}s_{i}\xi_{i},\;s_{i}>0,\\ y_{i}\left(wz_{i}+b_{i}\right)\geq 1-\xi_{i},\;\xi_{i}\geq 0,\;\;i=1,2,\ldots ,N. \end{array}$$

In (1), *C* is the penalty factor, *ξ*_*i*_ is the relaxation factor, and *s*_*i*_ is the coefficient of Lagrange.

The optimal hyperplane can be obtained by quadratic optimization. When the number of features is extremely huge, in order to solve the objective function effectively, we can transform the objective function into the corresponding dual forms.

Let the optimal solution be w*; thus the discriminate function for binary classification is defined as

(2) $$\begin{array}{c} f\left(x\right)=\mathrm{sgn}\left(\overset{N}{\underset{i=1}{\sum }}w_{i}^{*}y_{i}z_{i}+b^{*}\right). \end{array}$$

When we construct the optimal hyperplane in the feature space *H*, the training algorithm only uses the dot product in the space, Φ(*x*_*i*_) · Φ(*x*_*j*_). So, the only thing we need to do is finding a function *K* which satisfies *K*(*x*_*i*_, *x*_*j*_) = Φ(*x*_*i*_) · Φ(*x*_*j*_). In this way, we only need to operate the dot product which can be realized by the function *K* in the original space, and there is no need to know the form of the transformation Φ. According to the related functional theory, if and only if *K*(*x*_*i*_, *x*_*j*_) satisfies the Mercer constraint [20], the kernel function *K*(*x*_*i*_, *x*_*j*_) would be corresponding to an inner dot product in some transformation space.

Therefore, though introducing the kernel function, the discriminate function for binary classification is redefined as

(3) $$\begin{array}{c} f\left(x\right)=\mathrm{sgn}\left(\overset{N}{\underset{i=1}{\sum }}w_{i}^{*}y_{i}K\left(x_{i},x\right)+b^{*}\right), \end{array}$$

where b*is any *w*_j_* which satisfies the constraint *C* > *w*_j_* > 0. Putting it into (3), we obtain

(4) $$\begin{array}{c} y_{j}\left(\overset{l}{\underset{i=1}{\sum }}w_{i}^{*}y_{i}K\left(x_{i},x\right)+b^{*}\right)=1. \end{array}$$

### 2.3. Liver Cancer Recognition Based on SVM-IOMIL

**Figure 2:**
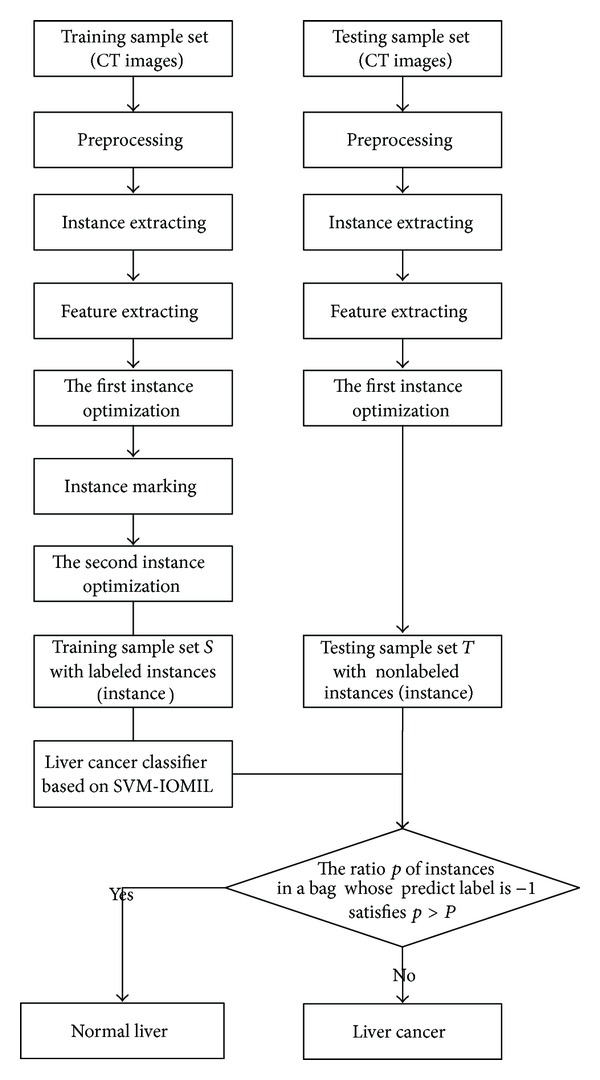
The main flow of the proposed method.

In this section, firstly we will introduce the whole procedure of the proposed method, which is shown in Figure 2, and then we will give a detailed explanation of each process.

In this paper, SVM-IOMIL_i_  (*i* = 1,2, 3) stands for three classifiers used for different datasets. SVM-IOMIL_1_ is a classifier for liver cancer and normal liver, SVM-IOMIL_2_ is a classifier for liver cancer and liver cirrhosis, and SVM-IOMIL_3_ is a classifier for liver cancer and liver cyst.

The process of the proposed method is as follows.

1. *Image Preprocessing.* Before the preprocessing, we extract the liver region manually from the abdominal CT image. Then we normalize the images and process them with histogram equalization after extraction.
2. *Instance Extracting.* We regard the liver CT image extracted by the first process as a bag and the block extracted equally based on the entire liver CT image as an instance.
3. *Feature Extraction*. In this paper, we use Gray Level Concurrence Matrix (GLCM) to extract the features for classification.
4. *Instance Optimization.* We use two-time instance optimization and CPSO-SVM to extract the instances and get the key instances finally.
5. *Predicting the Unknown Samples*. We use the key instances and a classification threshold to predict the unknown samples.

#### 2.3.1. Preprocessing

**Figure 3:**
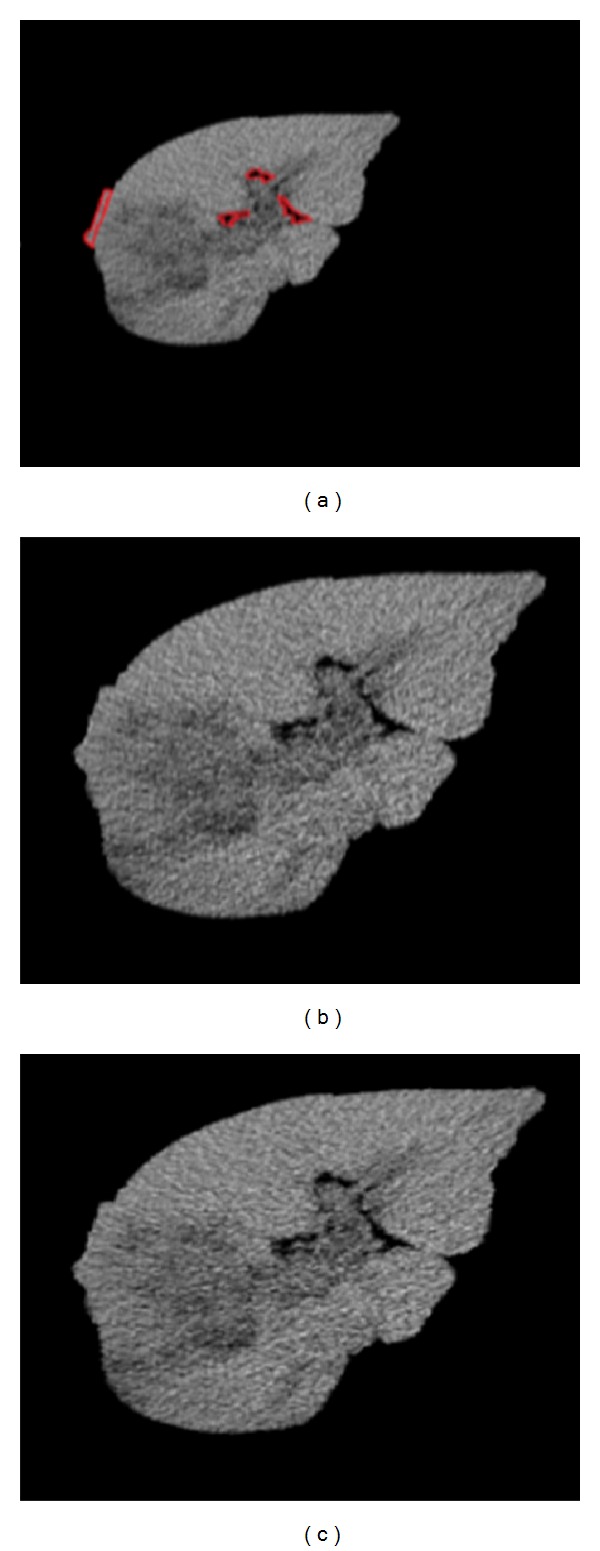
Preprocessing for liver CT images. (a) Liver region. (b) Normalization result. (c) Histogram equalization result.

Preprocessing includes image normalization and histogram equalization. As MIL algorithm does not require segmentation accuracy, we extract the liver region manually. The inaccurate sections of liver region are marked with a red curve in Figure 3(a).

In the procedure of image normalization, according to the location of the liver in the image and the experimental observation, we normalize the size of the images to 339 × 339. The result of the image after normalization is shown in Figure 3(b).

In order to make the image texture characteristics clearer, we process the CT images with histogram equalization, as a result we highlight the difference between two categories of images. The result of histogram equalization is shown in Figure 3(c).

#### 2.3.2. Instance Extraction

**Figure 4:**
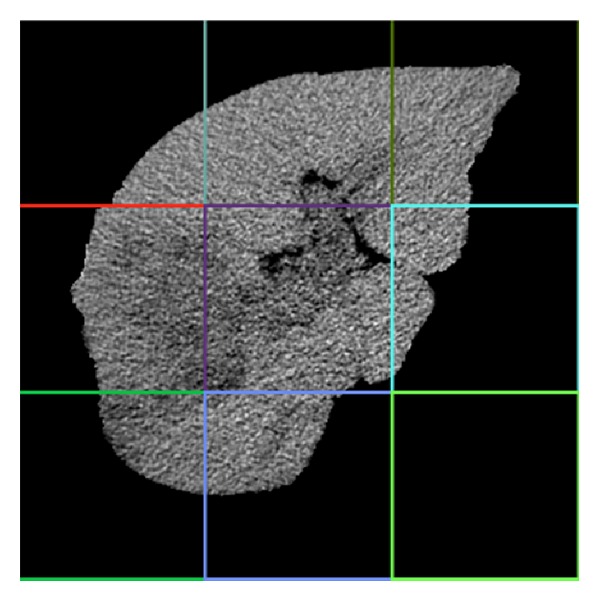
Bag structure sketch.

In order to extract the instances, firstly we define the CT image after preprocessing as a bag, and then we define the bag structure through extracting the blocks equally based on the entire liver CT image and define each block as an instance. The bag structure is shown in Figure 4.

The tumor block will be defined as a liver cancer instance, while the others will be defined as nonmalignant liver instances. Therefore, a nonmalignant liver instance can exist in a liver cancer image or a nonmalignant liver image, while a liver cancer instance can only exist in a liver cancer image. The bag is defined as positive if there is at least one liver cancer instance in the CT image. Otherwise it will be defined as negative.

#### 2.3.3. Feature Extraction

Angular second moment (ASM) can reflect an image's uniformity degree of grayscale distribution and the texture roughness. Entropy (ENT) can show the texture complexity of an image. Contrast (CON) can reflect the sharpness of the image and the depth of groove in texture. Correlation (COR) can show the correlation of local grayscale in an image. Therefore, we extract features from 8 matrices which are from 4 directions *θ* = {0°, 45°, 90°, 135°} and 2 distances *d* = {1,2} by Gray Level Concurrence Matrix (GLCM) for each instance. Finally we choose the mean and variance of 4 texture features, which are ASM, ENT, CON, and COR, respectively, in 2 distances as the experiment features. The design equations for the texture features are shown as

(5) $$\begin{array}{c} \text{ASM}=\underset{i}{\sum }\underset{j}{\sum }I{\left(i,j\right)}^{2},\\ \text{ENT}=-\underset{i}{\sum }\underset{j}{\sum }I\left(i,j\right)\text{lg}I\left(i,j\right),\\ \text{CON}=\underset{i}{\sum }\underset{j}{\sum }{\left(i-j\right)}^{2}I\left(i,j\right),\\ \text{COR}=\frac{\left[\sum_{i}\sum_{j}\left(\left(ij\right)I\left(i,j\right)\right)-u_{x}u_{y}\right]}{\sigma_{x}\sigma_{y}}, \end{array}$$

where *I*(*i*, *j*) is the element of the image, and *u*_*x*_,  *u*_*y*_,  *σ*_*x*_,  *σ*_*y*_ is defined as follows, respectively: *u*_*x*_ = ∑_*i*_∑_*j*_ *I*(*i*, *j*), *u*_*y*_ = ∑_*i*_ *i*∑_*j*_ *I*(*i*, *j*), *σ*_*x*_ = ∑_*i*_(*i* − *u*_*x*_)∑_*j*_ *I*(*i*, *j*), and *σ*_*y*_ = ∑_*j*_(*j* − *u*_*y*_)∑_*i*_ *I*(*i*, *j*).

#### 2.3.4. Instance Optimization

Before marking the instances, in order to minimize the interference of background and reduce the cost of operation, we optimize the instances for the first time. After marking the instances, in order to improve the classification performance, we optimize the instances for the second time.

*(1) The First Instance Optimization.* The ASM value of the background block, which is the block without liver region, is 1, while the ASM value of the block with the liver region cannot be 1. So, according to the ASM value, we can determine whether the current instance is background block or not. If it is background block, we abandon the instance directly to remove interference or we reserve it temporarily.

**Figure 5:**
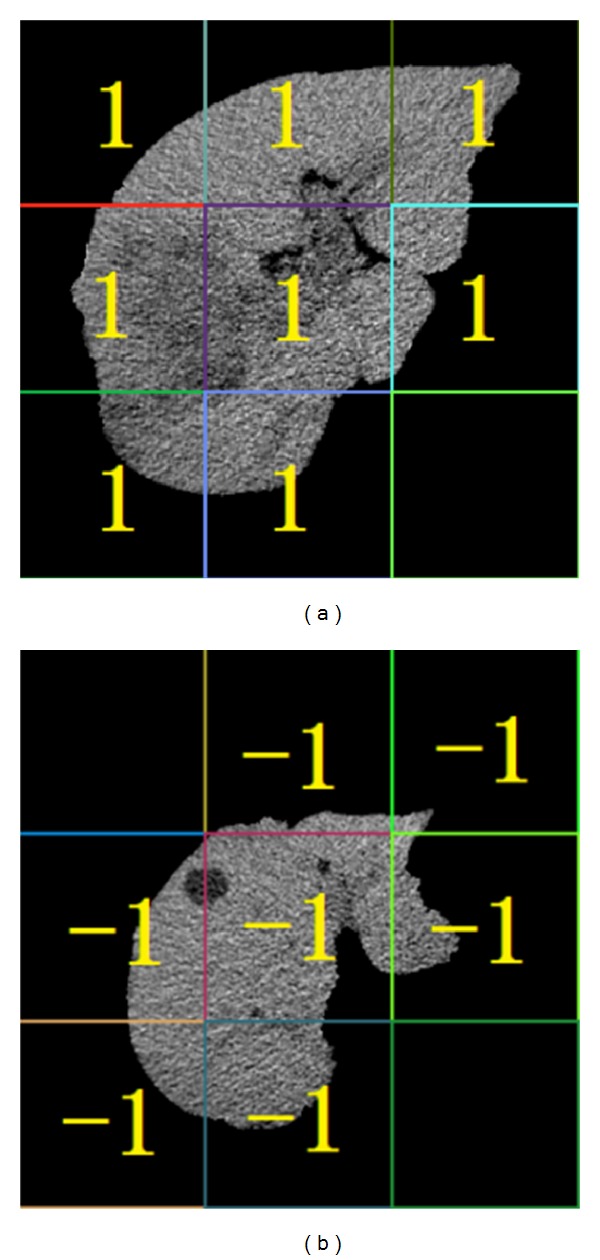
Result of the first instance optimization. (a) Instance label for liver cancer image. (b) Instance label for liver cyst image.

We labeled the instance with the label of the bag which the instance belongs to. Furthermore if the instance reserved after the first instance optimization is in a positive bag, we mark it to 1; otherwise, we mark it to −1. After the first instance optimization, the image with instance label is shown in Figure 5.

**Figure 6:**
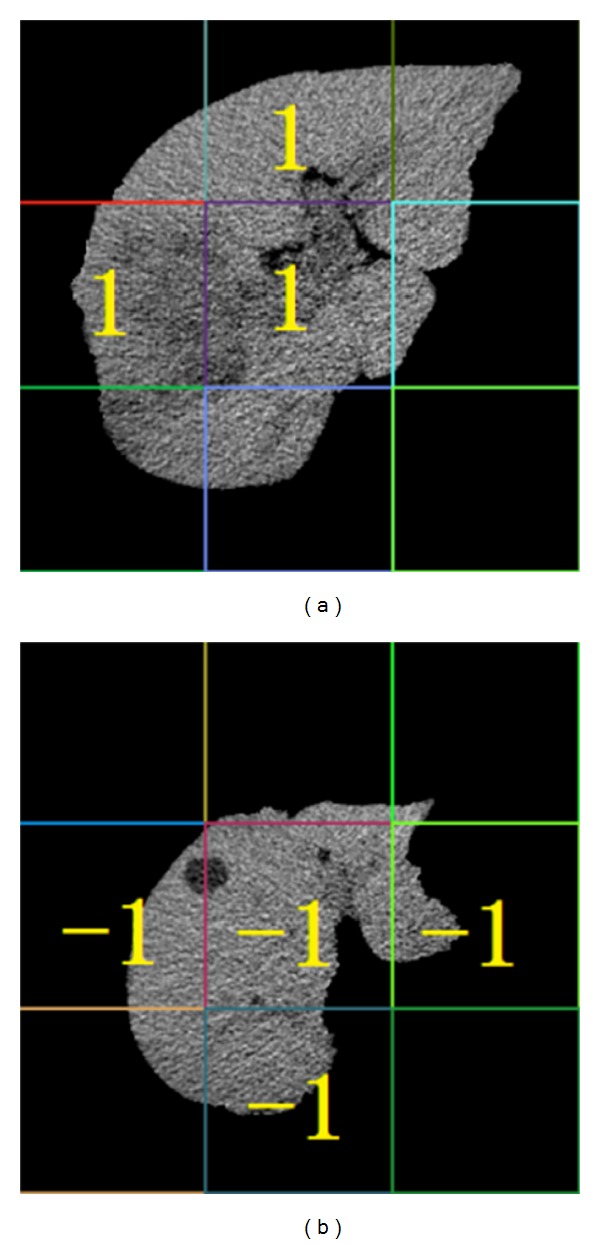
Result of the second instance optimization. (a) Instance label for liver cancer image. (b) Instance label for liver cyst image.

*(2) The Second Instance Optimization.* Figure 5 illustrates that some instances have less liver region and more background. This leads to the classification interference. So we improve the algorithm further. Firstly, in training phase, we choose the instances classified correctly in the bags which have the higher classification accuracy as “excellent instances.” Secondly, we store the “excellent instances” into a new training set. Then we determine the category of instances and bags according to the new training set. In this way, we have improved the accuracy of recognition. The second instance optimization must be processed through experiments, so we do not know the result in advance.  Figure 6 shows a possible case.

#### 2.3.5. Construction of Liver Cancer Classifier

*N* is the total number of instances and *N* = ∑_*i*=1_^*l*^ *n*_*i*_, *n*_*i*_ is the amount instances in *i*th bag. According to the character of liver cancer, we redefine the objective function for SVM as

(6) $$\begin{array}{c} \min L\left(w\right)=\frac{1}{2}{||w||}^{2}+C\overset{N}{\underset{i=1}{\sum }}a_{j}\xi_{i},\;a_{j}\in \left\{0,1\right\},\\ y_{i}\left(w+b_{i}\right)\geq 1-\xi_{i},\;\xi_{i}\geq 0,\;\;i=1,2,\ldots ,N, \end{array}$$

where *C* is the penalty factor. *ξ*_*i*_ is the relaxation factor. *a*_*j*_ is the parameter used for instance extraction and its default value is 0.

The process of classifier construction for liver cancer and normal liver is as follows:

 input: the training set *D* which contains the labeled training bags;
 output: SVM-IOMIL_1_ classifier (w*, b*).

**Step 1**

Set the instances attribute space *S* = *∅* and then extract instances in *S*.

**Step 2**

For all *B*_*i*_ ∈ *D*, *B*_*i*_ is a bag and *B*_*ij*_ is the *j*th instance of the bag *B*_*i*_.

If *B*_*i*_ is a positive bag, we classify the instance *B*_*ij*_ reserved after the first instance optimization in this bag, by SVM classifier. We label the “excellent instance” to 1, set the parameter *a*_*j*_ to 1, and add them to *S*.

If *B*_*i*_ is a negative bag, we classify the instance *B*_*ij*_, and then we label the “excellent instance” to −1, set the parameter *a*_*j*_ to 0, and add them to *S*.

**Step 3**

Optimize the parameters of the SVM classifier by choosing the best penalty factor *C* and the kernel function's control factor *g*.

**Step 4**

Set *S* as the training sample set and train the SVM-IOMIL_1_ classifier (w*, b*) according to (6).

**Step 5**

If *B*_*i*_ is not the last instance, then go to Step 2. Otherwise, we get our classifier and the key instances which is a set of the “excellent instances.”

The SVM-IOMIL_2_ classifier for liver cancer and liver cirrhosis and the SVM-IOMIL_3_ classifier for liver cancer and liver cyst are the same as SVM-IOMIL_1_, so there is no need to repeat them here.

#### 2.3.6. Prediction Algorithm for Classification

After the classifier construction, we use the liver CT images which are not used in training to test the classifier as follows:

 input: training sample set *S* and testing sample set *T* which contains the unlabeled test bags;
 output: the classification result of the test bag *B*.

**Step 1**

Put the test bag *B* contained in testing sample set *T* into SVM-IOMIL_1_ classifier (w*, b*).

**Step 2**

Predict the reserved instance after the first instance optimization in test bag *B* according to (3).

If the ratio that the label of the instance in test bag *B* predicted to be −1 is larger than threshold *P*, the test bag *B* will be a negative bag; otherwise, it will be a positive bag.

## 3. Results and Discussion

### 3.1. The Experimental Data and Environment

The original data are 440 abdominal CT images provided by the radiology department of a large hospital in Shenyang, China. These images are abdominal CT images with a resolution of 512 × 512 pixels, which are BMP format. After preprocessing, the images we use in the experiments are liver CT images with a resolution of 339 × 339 pixels and their format is BMP. The images include 120 normal liver cases, 120 liver cyst cases, 120 liver cirrhosis cases, and 80 liver cancer cases. The datasets used in this paper are shown in Table 1.

In this paper, we divide each kind of the images into two parts randomly and equally. One part is regarded as a potential sub-set of the training set, and the other one is the part of the potential subset of the testing set. The training set consists of one part of the liver cancer images and one part of other images. We divide the original testing data randomly into 5 groups and then we regard the data in one group as current validation set and the data of the key instances as training set. Finally, we use the average value of the validation set's evaluation criterion in these 5 groups as the classifier's performance evaluation criterion.

Experimental environment is as follows: Intel(R) Core (TM) i7-2600 CPU @3.4 GHz, 4 G RAM, 900 G hard disk, Windows7 OS, and MATLAB 7.14 simulation environment.

### 3.2. Evaluation Criterion for Classification Performance

In experiments, we use accuracy (ACC), sensitivity (SEN), specificity (SPE), processing time (PT), and training time (TT) to evaluate classification performance of the liver cancer recognition experiment. The definitions of the evaluation criterion are shown as

(7) $$\begin{array}{c} \text{ACC}=\frac{\left(\text{TP}+\text{TN}\right)}{\left(\text{TP}+\text{FN}+\text{TN}+\text{FP}\right)},\\ \text{SEN}=\frac{\text{TP}}{\left(\text{TP}+\text{FN}\right)},\\ \text{SPE}=\frac{\text{TN}}{\left(\text{TN}+\text{FP}\right)}, \end{array}$$

where TP and FN are the number of positive samples discriminated right and wrong, respectively, and TN and FT are the number of negative samples discriminated right and wrong, respectively.

Sensitivity mainly represents the recognition accuracy of liver cancer. Specificity represents the recognition accuracy of nonmalignant liver.

 Processing time (PT): the time from inputting images to extracting features.
 Training time (TT): the time from acquiring the information of feature data to acquiring the result of classification.

### 3.3. Experimental Results and Analysis

#### 3.3.1. Determining the Best Block Length

**Figure 7:**
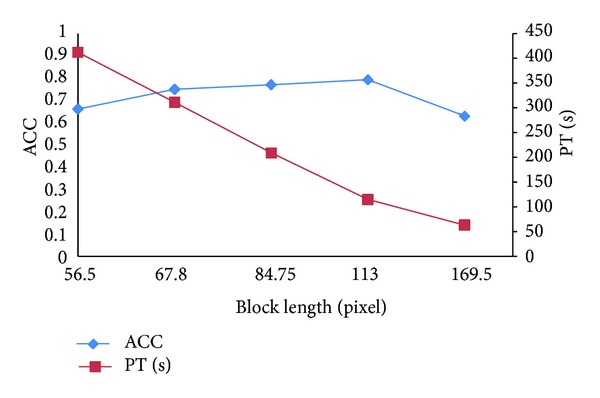
Classification results on different block length.

When we regard the blocks which we segmented equally based on the entire liver CT image as instances, the size of the block has a great influence on classification results. In order to obtain an objective data, we do multigroup experiments on different block length based on the existing Citation-kNN algorithm. The experimental results are shown in Figure 7.

Analyzing the experimental data from Figure 7, we obtain the following conclusions. (1) The smaller the size of block is, the more the number of instances is and the more the PT is, but the effect on ACC is small. (2) When the size of blocks is small, with the block length increasing, ACC increases. When the size of blocks is bigger than 113, with the block length increasing, ACC decreases. When the size of block is 169.5, ACC decreases sharply.

The reasons are as follows. (1) The more blocks lead to more time for feature extraction, so the PT increases. (2) Using a smaller block length means fewer pixels in the block, and GLCM is statistics-based texture features. Therefore it does not reflect the statistical properties. Furthermore the speckle noise is also easy to affect the quality of feature extraction and ultimately makes ACC decrease. After that, through the increasing block length, the amount of information in a block increases, and ACC gradually increases. But to a certain amount, since the block is too large, much more complex texture information will be mixed; ACC decreases instead. Considering PT and ACC, we obtain the best classification effect when the block length is 113.

#### 3.3.2. Determining the Threshold

**Figure 8:**
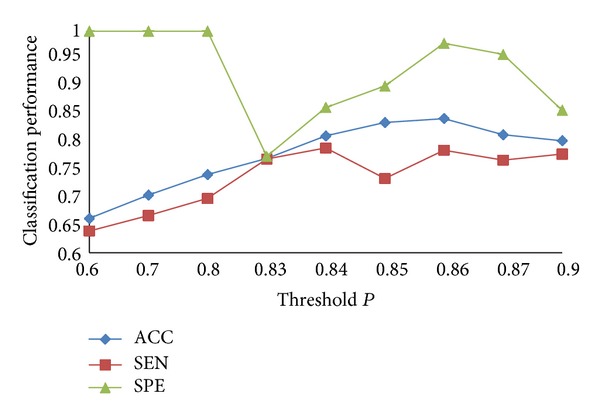
Classification results on different thresholds.

The instances in a liver cancer image are not all tumor blocks. In fact few of them are tumor blocks. So the threshold *P* should be in [0.5,1]. We do 9 groups of experiments with different threshold *P*, and the minimum increase margin is 0.01. The comparison of experimental results is shown in Figure 8.

In Figure 8, when *P* varies from 0.6 to 0.85, ACC increases obviously; when *P* is 0.86, ACC is the best; when *P* continues to increase, ACC decreases instead. As a whole SEN increases firstly and then decreases, SPE decreases firstly and then increases. They can achieve a balance when ACC is the best. The reasons are as follows. When *P* is smaller, it means that we regard more liver cancer cases as nonmalignant liver cases, and this will produce a large number of false positive bags. As a result, all the nonmalignant liver cases can be recognized correctly, but ACC of liver cancer cases is very low. When *P* is a certain value, we can recognize the liver cancer better. Through analysis of the experiment results, when *P* is 0.86, we get the best classification effect.

#### 3.3.3. Parameter Optimization for SVM

Parameter optimization undoubtedly can improve the accuracy of classification when we use SVM for classification. There are many parameters in SVM, and they always have a default value. But we cannot get the desired effect in many cases with the default values. So we optimize the parameters *C* and *g* in order to achieve the optimal classification results. Firstly, we get the best *C* and *g* when we classify the bag with “excellent instance” by PSO algorithm. Then we use LO to get the final optimal SVM parameters. The process of LO is as follows.

**Figure 9:**
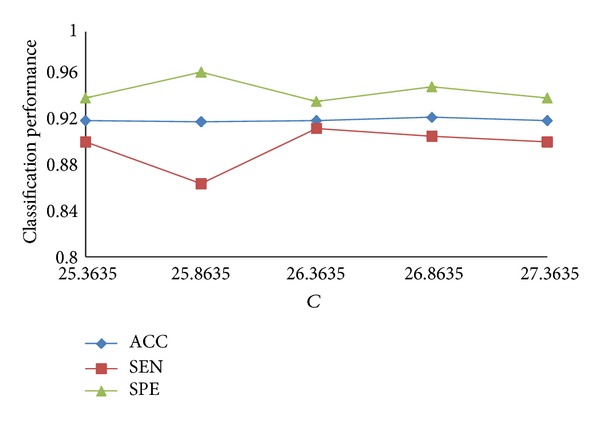
Classification results with different values of *C*.

**Figure 10:**
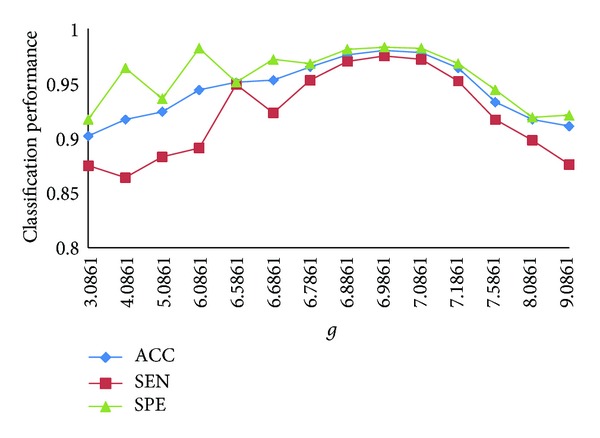
Classification results with different values of *g*.

The values of *C* generally focus on the scope of 25 to 27 in the bag with “excellent instance,” and *g* focus on the scope of 3 to 10. We choose one pair of the parameters, *C* = 26.3635  and  *g* = 5.0861, as the initial parameters.  Figure 9 shows the classification results with different values of *C* when *g* is 5.0861, and Figure 10 shows the classification results with different values of *g* when *C* is 26.8635.

In Figure 9, with the change of the value of *C*, the effect on ACC is not obvious, and the best *C* we choose is 26.8635. SPE and SEN are always one increased and another decreased, but they can achieve a better result relatively when ACC is the best.

In Figure 10, the value of *C* is 26.8635. When the value of *g* is increasing, ACC increases. When *g* is bigger than 6.9861, ACC decreases following the increasing of *g*. Thus, we obtain the best ACC when *C* is 26.8635 and *g* is 6.9861. Although SEN and SPE have some fluctuation, they achieve a satisfying result when ACC is the best.

#### 3.3.4. Experimental Results by SVM-IOMIL with Different Classification Samples

**Figure 11:**
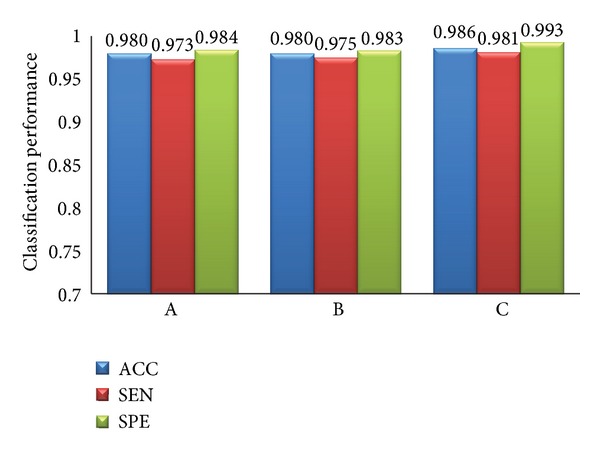
Classification results with different samples.

The classification sample sets are liver cancer and normal liver, liver cancer and liver cyst cases, and liver cancer and liver cirrhosis, and we represent them by A, B, and C, respectively. The experimental results are shown in Figure 11.

As we can see in Figure 11, the proposed classification algorithm has generality. It has a high ACC, SEN, and SPE for the three different samples. The ACCs for the three different samples are all over 98%. And the inputting of experimental data and processing of the three experiments are similar, so PT and TT are the same for different samples.

#### 3.3.5. Comparison between Our Algorithm and Other Algorithms

**Figure 12:**
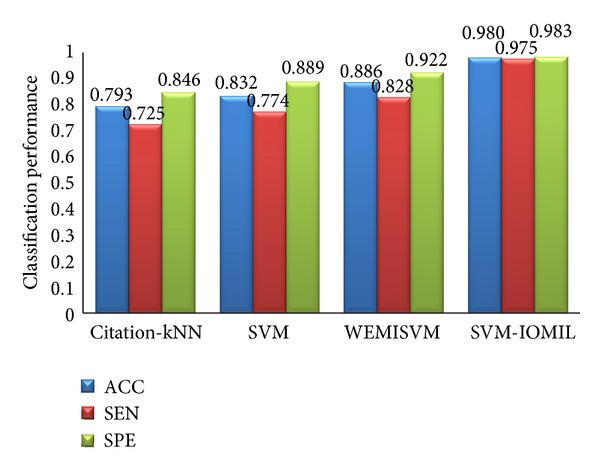
The classification efficiency comparison of four different algorithms.

**Figure 13:**
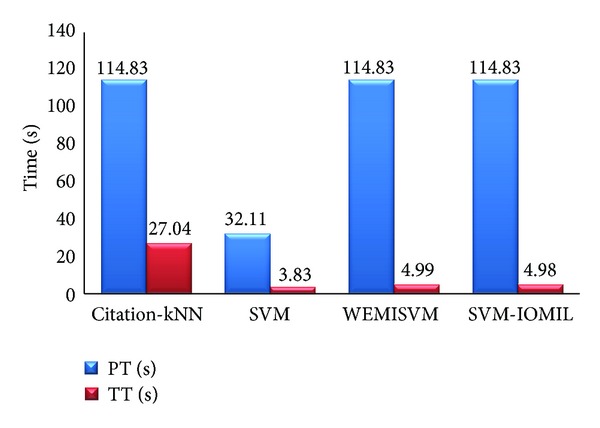
The time efficiency comparison of four different algorithms.

Several contrast experiments are carried out with the same feature data and different algorithms in this paper. The algorithms are Citation-kNN algorithm with minimum Hausdorff distance, the traditional SVM algorithm, WEMISVM algorithm, and our algorithm. The classification results of these algorithms are shown in Figures 12 and 13.

As Figure 12 shows, ACC, SEN, and SPE of our algorithm are higher than those of the other three algorithms obviously.

As for PT, the traditional SVM algorithm is much less than the other three algorithms. Citation-kNN algorithm, WEMISVM algorithm, and our algorithm are all MIL algorithms, while the SVM algorithm is a traditional single instance algorithm. The MIL algorithm needs to extract more instances, but the traditional algorithm needs one.

As a lazy learning algorithm, Citation-kNN algorithm needs to save the whole training set and go through the whole sample space when predicting, so it costs more time when classifying. The SVM algorithm, WEMISVM algorithm, and our algorithm benefit from the advantage of SVM, so they need less TT than Citation-kNN algorithm.

## 4. Conclusions

This paper proposed a novel MIL method to recognize liver cancer with abdominal CT images based on two-time instance optimization and CPSO-SVM. We eventually got three better classifiers to classify the liver cancer and the normal liver, the liver cancer and the liver cirrhosis, and liver cancer and the liver cyst. The proposed algorithm achieved a better classification accuracy and robustness of ROI segmentation. As the contrast experiments show, our method greatly improved the recognition accuracy for liver cancer. The instances are extracted equally based on the entire image, so our method can ignore the process of tumor region segmentation and lower the requirements of liver region segmentation accuracy. However, the processing speed of our algorithm is lower than traditional SVM algorithm because our algorithm is a MIL algorithm. Obviously, MIL algorithm will extract features for more objects than the traditional single instance classification algorithm, so it certainly needs more time for image processing. This is also the main problem of MIL algorithm at present. Besides, our algorithm is only used in binary classification problems. In the future, we will explore some methods to reduce the time complexity of the MIL algorithm and come up with a new classification method to solve the multiclassification problems.

## Figures and Tables

**Table 1:** Datasets used in our experiments.

Images	Training samples	Testing samples	Total
Liver cancer	40	40	80
Normal cancer	60	60	120
Liver cirrhosis	60	60	120
Liver cyst	60	60	120
